# Usefulness of the SARC-F questionnaire and the measurement of the hand grip strength in predicting short-term mortality in older patients hospitalized for acute heart failure

**DOI:** 10.1007/s41999-024-01054-2

**Published:** 2024-09-27

**Authors:** Chukwuma Okoye, Virginia Morelli, Riccardo Franchi, Tessa Mazzarone, Daniela Guarino, Lorenzo Maccioni, Cristina Cargiolli, Valeria Calsolaro, Filippo Niccolai, Agostino Virdis

**Affiliations:** 1 School of Medicine and Surgery, University of Milano-Bicocca, Piazza dell’Ateneo Nuovo 1, 20126 Milano, MI Italy; 2 Acute Geriatrics Unit, Fondazione IRCCS San Gerardo dei Tintori, Monza, Italy; 3 Geriatrics Unit, Department of Clinical & Experimental Medicine, University Hospital of Pisa, Pisa, Italy; 4 Primary Care Unit, Azienda ASL Toscana Nord Ovest, Lucca, Italy

**Keywords:** Heart failure, Older adults, Sarcopenia, Handgrip, Outcomes

## Abstract

**Purpose:**

Sarcopenia is a potentially reversible syndrome that increases the risk of cardiogenic cachexia and adverse outcomes in older patients with heart failure (HF). Despite its clinical significance, sarcopenia remains underdiagnosed due to the complexities of comprehensive assessment in patients with acute HF. This study aimed to evaluate whether the SARC-F questionnaire, its inviduals components, and the handgrip strength test (HGS) can predict short-term prognostic risk in very old patients recently discharged after acute HF.

**Methods:**

We consecutively enrolled patients aged 75 years or older hospitalized with acute HF in the Geriatrics Unit of a tertiary care hospital. All patients underwent physical examination, complete blood tests, point-of-care ultrasound, and a comprehensive geriatric assessment, including physical performance through SARC-F and HGS. The thirty-day post-discharge mortality rate was assessed by phone interview.

**Results:**

Out of 184 patients hospitalized with acute HF who were enrolled in the study (mean [SD], 86.8 [5.9] years, 60.3% female), 47 died within 30 days after discharge. By multivariate logistic analysis, HGS (*β* = – 0.73 ± 0.03, *p* = 0.008) and SARC-F [adjusted OR = 1.18 (CI 95% 1.03–1.33), *p* = 0.003] resulted independently associated with mortality. Furthermore, two SARC-F sub-items, namely, limitation in rising from a chair and history of falls [aOR: 3.26 (CI95% 1.27–8.34), *p* = 0.008; aOR: 3.30 (CI 95% 1.28–8.49), *p* = 0.01; respectively] emerged as determinants of 30-days mortality.

**Conclusion:**

SARC-F and HGS test independently predict 30-day post-discharge mortality in oldest-old patients hospitalized for acute HF.

**Supplementary Information:**

The online version contains supplementary material available at 10.1007/s41999-024-01054-2.

## Key summary points

**Aim:**

To determine the usefulness of the SARC-F questionnaire and the Hand grip strength test (HGS) in predicting short-term mortality among hospitalized older patients with acute HF.

**Findings:**

SARC-F, its components, and the HGS test were associated with 30-day post-discharge mortality in older patients with acute HF.

**Message:**

SARC-F and HGS can reliably predict short-term mortality in older patients with acute HF.

**Supplementary Information:**

The online version contains supplementary material available at 10.1007/s41999-024-01054-2.

## Introduction

Heart failure (HF) is one of the major causes of mortality and hospitalization worldwide, and 4 out of 5 patients with HF are older than 65 years [1]. Despite medical improvement, the five-year survival rates for HF remain poorer than most cancers [2]. In particular, the risk of re-hospitalization or death is highest in the 30 days following discharge for acute decompensated heart failure (ADHF) [3]. Moving the aim away from the treatment of a single disease and toward a more holistic approach, international guidelines advocate for a comprehensive multidisciplinary assessment of HF patients[4]. In this regard, recent investigations reported that approximately 30% of patients with HF may have a diagnosis of sarcopenia [5], a potentially reversible geriatric syndrome characterized by a progressive loss of skeletal muscle and strength resulting in impaired physical performance that can gradually lead to disability, reduced quality of life, and even death [6, 7]. Compared to non-HF patients, those with HF have a significantly higher prevalence of sarcopenia [8] thus suggesting the existence of a strong interconnection between the pathophysiological pathways involved in HF, age-related changes in body composition, and sarcopenia [9]. Patients with HF and sarcopenia are more prone to develop physical frailty and related negative health outcomes, with a higher risk of HF progression with the need for hospitalization [7] and an increased mortality rate [10–12]. While in the FRAGILE HF study, the prevalence of sarcopenia was 22.7% [13], a recent meta-analysis reported a 43% sarcopenia prevalence among patients with acutely decompensated HF[5], shedding light on an under-recognized syndrome in hospitalized older patients. Sarcopenia and its components have been proven to be associated with all-cause mortality[14]; notwithstanding, very few studies have attempted to evaluate the short-term prognostic significance of sarcopenia in older individuals with acute HF [13, 15–18]. According to the European Working Group on Sarcopenia in Older People 2 (EWGSOP2) guidelines [8], all patients with suspected sarcopenia should undergo the SARC-F, a 5-item questionnaire that is self-reported by patients based on their perception of limitations in strength, walking ability, rising from a chair, stair climbing, and experiences with falls. Subsequently, several diagnostic evaluations should be made to correctly diagnose sarcopenia, namely: (i) evaluation of muscle strength using a calibrated handheld dynamometer or chair stand test, (ii) assessment of muscle quantity through magnetic resonance imaging (MRI), computer tomography (CT) or Dual-energy X-ray absorptiometry (DEXA), (iii) recognition of low physical performance that can be measured by gait speed, Short Physical Performance Battery (SPPB) [19] and the Timed-Up and Go test (TUG). Nevertheless, SARC-F is an inexpensive and convenient method for sarcopenia risk screening [20] and has been proven valid and accurate for identifying people at risk of sarcopenia-associated adverse outcomes [21]. Given these premises, SARC-F could be helpful in predicting the outcomes of older patients with ADHF. This study aimed to determine the short-term prognostic role of the SARC-F questionnaire in hospitalized older patients with ADHF. The secondary endpoint was to evaluate the relationship between in-hospital Hand Grip Strength test (HGS) values, SARC-F sub-items, and short-term prognosis.

## Methods

We prospectively enrolled all patients aged 75 years or older hospitalized with acutely decompensated HF in our tertiary care hospital between January 1, 2022, and February 1, 2023. The exclusion criteria were: (1) inability to communicate with researchers or obtain informed consent; (2) critical illness requiring invasive ventilation at admission (3) acute coronary syndrome; (4) inability to perform an HGS test.

A panel of clinicians adjudicated the diagnosis of congestive heart failure based on clinical symptoms, signs, chest x-ray film results, echocardiographic findings, and therapy at admission in line with recent international guidelines [4]. All patients underwent a focused cardiac ultrasound (FOCUS), performed by an expert clinician. FOCUS follows the principle formulated in the focused assessed transthoracic echocardiography (FATE) protocol [22]. Furthermore, as patients underwent a diagnostic examination with bedside Point of care ultrasound (POCUS) including lung ultrasound, focused cardiac ultrasound, pleural effusion score (PEFS) [23], and inferior vena cava (IVC) assessment. All the patients underwent physical examination, complete blood tests, and a comprehensive geriatric assessment (CGA) [24] including cognitive evaluation using the Short Portable Mental Status Questionnaire (SPMSQ) [25], calculation of the burden of comorbidities through the Cumulative Illness Rating Scale – Comorbidity Index, (CIRS-CI), level of autonomy in terms of independence in the performance of basic (ADL) [26], and instrumental (IADL) [27] activities of daily living and frailty assessment through the Clinical Frailty Scale (CFS) [28]. Systolic Blood Pressure (SBP) and Heart Rate (HR) were also collected at hospital discharge. SBP was measured using an automated electronic blood pressure device (OMRON X3 Comfort, HEM-7155-EO, Omron Co, Kyoto, Japan). Measurements were taken with the patient in a seated or semi-seated position. Three readings were taken at one-minute intervals to ensure accuracy. Measurements were taken on the left arm unless contraindicated (e.g., due to a medical condition affecting the left arm), in which case the right arm was used.

The risk of malnutrition was assessed through the Mini Nutritional Assessment-Short Form (MNA-SF) [29] and Body Mass Index (BMI). Functional capabilities and physical performance were evaluated through a pre-morbid SARC-F and HGS. SARC-F includes 5 items: strength, assistance in walking, rising from a chair, climbing stairs, and history of falls. Each component is scored on a 0–2 scale and summed for an overall range of 0–10. A total score of SARC-F ≥ 4 indicates a high risk of sarcopenia [30]. Given that the onset of acute HF could influence physical performance, patients or caregivers were asked to answer SARC-F questions regarding the physical status and habits approximately 30 days prior to hospital admission for heart failure (pre-morbid status). The HGS test was performed using a hand dynamometer (CAMRY EH-101, Sensun Weighing Apparatus Group Ltd, Guangdong, China) with the dominant hand. HGS is a simple measure of strength and may be utilized as a marker of mobility. The cut-off level of < 16 kg in women and < 27 kg in men has been identified to detect patients at risk for sarcopenia. Participants were seated with shoulder adducted, elbow flexed to 90 degrees, and forearm and wrist neutral. The highest score of three consecutive measurements was recorded. The 30 post-discharge mortality rate was assessed by phone interview.

### Statistical analysis

Statistical analysis was performed with IBM SPSS Statistic (IBM SPSS Statistic version 27.0 lnk IBM Corporation and its licensor 1989–2020) and RStudio (RStudio Team: Integrated Development for R. RStudio, PBC, Boston, MA). Continuous variables were presented as mean and standard deviation, ordinal variables as median and interquartile range (IQR), and categorical variables as percentage. Mann–Whitney and chi-square tests were used for multiple comparisons.

A multivariable logistic regression was performed to evaluate the association between SARC-F (as an ordinal variable, ranging from 0 to 10), HGS test, and 30-days-mortality using a priori selected model covariates based on clinical considerations from our previous study [31] and variables statistical significance. Directed Acyclic Graph was performed to identify potential confounders and the Variance Inflation Factor (VIF) was calculated to check for multicollinearity. Covariates included in the multivariable model were age, sex, number of comorbidities, NT-pro-BNP, and SBP at discharge. As a secondary analysis, a logistic multivariable analysis was performed among SARC-F sub-items with a result statistically significant at the univariate analysis. The number of falls was categorized as (0): no falls, used as reference, (1) 1–3 falls in the last year (2): greater than or equal to four falls. Estimate odds ratios (ORs) with 95% confidence intervals (CIs) were obtained. Finally, a Spearman’s rank correlation Rho was performed to evaluate to verify the relationship between SARC-F and HGS. The sample size was calculated on the basis of previous research and required at least 155 patients, to detect a 10% difference in the odds ratio for a 1-point increase of SARC-F (as ordinal predictor), with a power of 80% and a 0.05 α risk. Tests were performed considering a level of significance of 5%.

## Results

As shown in Supplementary Fig. 1, 229 patients hospitalized with acute HF were assessed for eligibility. Thirty-two patients were not included in the study due to in-hospital deaths (10), inability to participate (19), or refusal (3). Thus, 191 patients were finally enrolled, of whom 7 were lost to follow-up. The remaining 184 patients (mean [SD], 86.8 [5.9] years) were ultimately included in the statistical analysis; among them, 47 (25.5%) died within 30 days following hospital discharge. No differences were found between deceased and survived patients in terms of gender prevalence, mean age, body weight, or the number and type of comorbidities (see Table 1 and Supplementary Tables 1, 2). Deceased patients had a higher median SARC-F [8 (IQR = 3.5) vs 5 (IQR = 6); respectively, *p* = 0.005] and mean Hand Grip Strength [11.1 (SD = 7.4) Kg vs 15.8 (SD = 7.7) Kg, respectively; *p* = 0.006], compared to their counterparts. After stratifying by sex, we observed that only 10 (13.3%) out of 73 males had HGS levels higher than 27 kg and, similarly, 27 (22.6%) out of 119 females performed an HSG test higher than 16 kg (Supplemental Tables 3 and 4). Regarding pulmonary and systemic congestion, no differences were detected in terms of B lines number, pleural effusion score (PEFS), and characteristics of inferior vena cava (Supplemental Table 1).

**Table 1:** Comparison between deceased patients and controls

	All patients *N* = 184	Dead *N* = 47	Alive *N* = 137	*P* value
Female (%)	111 (60.3)	28 (59.6)	83 (60.6)	0.90
Age mean, years (SD)	86.8 (5.9)	87.6 (6)	86.6 (6)	0.30
BMI mean, kg/m^2^ (SD)	24.6 (4.5)	24.1 (4.5)	24.8 (4.6)	0.49
HGS mean, kg (SD)	14.8 (7.8)	11.1 (7.4)	15.8 (7.7)	**0.001**
HGS mean, kg (SD), F	11.5 (5.5)	7.5 (5.2)	12.6 (5.1)	**0.002**
HGS mean, kg (SD), M	19.3 (8.4)	15.7 (7.4)	20.4 (8.5)	**0.035**
CFS median (IQR)	6 (3)	7 (1)	5 (3)	** < 0.001**
SARC-F median (IQR)	5 (5)	8 (3.5)	5 (6)	**0.005**
SARC-F ≥ 4 (%)	122 (66.1)	37 (79)	85 (62)	**0.04**
Length of stay median, days (IQR)	7 (3)	8 (4)	7 (4)	0.06
Number of medications median (IQR)	8 (3)	8 (3)	9 (3)	0.60
N° of comorbidities median (IQR)	4 (2.5)	4 (2.5)	4 (2)	0.29
CIRS – CI median	3 (2)	3 (1.2)	3 (2)	0.06
LVEF < 40% (%)	58 (31.7)	15 (31.4)	43 (31.8)	0.97
LVEF %, median	52 (10)	53 (17.5)	52 (15.5)	0.68
Mean SBP (SD)	123 (21)	116 (20)	126 (21)	**0.044**
NT-proBNP median, pg/mL (IQR)	3843 (6897.5)	6443 (11,455)	3505 (5615.5)	**0.03**
Mean Creatinine (SD)	1.39 (0.71)	1.49(0.84)	1.36(0.65)	0.395
Albumin mean, g/dl (SD)	3.03 (0.53)	2.9 (0.55)	3.1 (0.51)	0.19
Mean Hemoglobin g/L (SD)	10.7 (1.8)	10.4 (1.9)	10.8 (1.7)	0.318
P/F admission mean (SD)	311.3 (93.7)	308.3 (100.5)	312.3 (91.6)	0.80

Mann–Whitney test, Pearson’s Chi-squared test or Fisher Test as appropriate. *P* values < 0.05 are in bold

*BMI* indicates Body Mass Index, *HGS* Hand Grip Strength, *CFS* Clinical Frailty Scale, *F* Female sex, *M* Male sex *CIRS-CI* Cumulative Illness Rating Scale – Comorbidity Index, *SARC-F* Strength- Assistance with walking- Rising from a chair- Climbing stairs and Falls questionnaire, *LVEF* Left Ventricular Ejection Fraction, *NT-proBNP* N-Terminal pro-B-type Natriuretic Peptide, *SBP* Systolic blood pressure, *P/F* PaO_2_/FiO_2_ ratio

As shown in Table 2, 122 patients (66.3%) were found to be at high risk of sarcopenia (SARC-F ≥ 4), showing a 1.9 times higher 30-day mortality than controls (30% vs. 16%, *p* = 0.04). Patients with SARC-F ≥ 4 were older [mean age: 87.8 years (SD = 5.5) vs 84.5 (SD = 6.2); respectively, *p* < 0.001] and more frequently women (65% vs. 48.3%, *p* = 0.03), and lower HGS score [mean HGS test: 12.9 (SD = 6.8) vs 18.9 (SD = 8.3) respectively; *p* < 0.001], compared to their peers. By Point-of-care Ultrasound (POCUS) assessment, we observed that patients at high risk of sarcopenia had higher Pleural Effusion Score [3 (IQR = 5) vs 1 (IQR = 4); respectively, *p* = 0.009] and a higher prevalence of valvular diseases compared to counterparts. Concerning comorbidities, we found a higher prevalence of chronic heart failure (68.4% vs. 50.8%, *p* = 0.02) in patients at high risk of sarcopenia compared to those at low risk (Supplemental Table 5 and 6).

**Table 2:** Comparison between patients with positive screening for sarcopenia (SARC-F ≥ 4) and controls

	All patients *N* = 184	SARC-F ≥ 4 *N* = 122	SARC-F < 4 *N* = 62	*P* value
Female (%)	111 (60.3)	81 (65)	30 (48.3)	**0.03**
Age mean, years (SD)	86.8 (5.9)	87.8 (5.5)	84.5 (6.2)	** < 0.001**
BMI mean, kg/m^2^ (SD)	24.6 (4.5)	24.1 (4.6)	25.6 (4.3)	0.07
HGS mean, kg (SD)	14.8 (7.8)	12.9 (6.8)	18.9 (8.3)	** < 0.001**
HGS mean, kg (SD), F	11.5 (5.5)	10.7 (5.2)	13.9 (4.6)	**0.002**
HGS mean, kg (SD), M	19.3 (8.4)	16.3 (7.3)	23.5 (8.3)	** < 0.001**
CFS median (IQR)	6 (3)	7 (2)	4 (2)	** < 0.001**
Hospital stay median, days (IQR)	7 (3)	7 (3)	7 (3.25)	0.60
N° of comorbidities median (IQR)	4 (2.5)	4 (3)	4 (2.25)	0.49
Number of medications median (IQR)	8(3)	9(4)	8(4)	0.11
CIRS – CI median	3(2)	3(2)	3(2)	0.31
LVEF < 40% (%)	58 (31.7)	25 (28)	15 (35)	0.36
LVEF %, median	52(10)	53(18)	50(15)	0.36
NT-proBNP median, pg/ml (IQR)	3843 (6897.5)	4257(6553.22)	2112 (4630.5)	0.20
Mean SBP (SD)	123 (21)	123 (22)	123 (19)	0.88
Mean Creatinine (SD)	1.39 (0.71)	1.45 (0.75)	1.26 (0.59)	0.21
Mean Hemoglobin g/L (SD)	10.7 (1.8)	10.5 (1.8)	11.3 (1.7)	0.05
P/F admission mean (SD)	311.3 (93.7)	316.3 (90.3)	298.9 (101.4)	0.26
30 days mortality (%)	47 (25.5)	37 (30)	10 (16)	**0.04**

Mann–Whitney test, Pearson’s Chi-squared test or Fisher Test as appropriate. *P* values < 0.05 are in bold

*BMI* indicates Body Mass Index, *HGS* Hand Grip Strength, *CFS* Clinical Frailty Scale, *F* Female sex, *M* Male sex *CIRS-CI* Cumulative Illness Rating Scale – Comorbidity Index, *SARC-F* Strength- Assistance with walking- Rising from a chair- Climbing stairs and Falls questionnaire, *LVEF* Left Ventricular Ejection Fraction, *NT-proBNP* N-Terminal pro-B-type Natriuretic Peptide, *SBP* Systolic Blood Pressure, *P/F* PaO_2_/FiO_2_ ratio

Regarding SARC-F items, as shown in Table 3, we observed a higher proportion of inability to rise from a chair in deceased patients compared to those survived (53.5% vs 29.2%, respectively, *p* = 0.01), and an increased number of falls in the last year prior hospitalization (1–3 falls in 30.2% vs 14.5%, respectively; 4 or more falls in 25.6% vs 14.5%, respectively, *p* = 0.01 for both); no other relevant differences were found across other items.

**Table 3:** SARC-F items and their relationship with 30-day post-discharge mortality

Sarc-F Items	All patients *n* = 184	Death 30 days *n* = 47	Alive *n* = 137	*P* value
Limitation in lifting 10 kg (%)				
None (0 pt)	34 (18.2)	8 (16.3)	26 (18.8)	0.45
Some (1 pt)	41 (22.2)	8 (16.3)	33 (24.1)	
Unable (2 pt)	109 (59.6)	31 (67.4)	78 (57.1)	
Limitation in walking across a room (%)				
None (0 pt)	65 (35.6)	11 (23.3)	54 (39.5)	0.11
Some (1 pt)	49 (26.5)	13 (27.9)	36 (26.1)	
Unable (2 pt)	70 (37.9)	23 (48.8)	47 (34.4)	
Limitation in rising from a chair (%)				
None (0 pt)	62 (33.9)	10 (20.9)	52 (38)	**0.01**
Some (1 pt)	57 (31.1)	12 (25.6)	45 (32.8)	
Unable (2 pt)	65 (35)	25 (53.5)	40 (29.2)	
Limitation in climbing 10 stairs (%)				
None (0 pt)	33 (18.1)	6 (13.9)	27 (19.4)	0.11
Some (1 pt)	56 (30.5)	10 (20.9)	46 (33.6)	
Unable (2 pt)	95 (51.4)	31 (65.2)	64 (47)	
N° of falls in last year (%)				
None (0 pt)	118 (64.7)	21 (44.2)	97 (71)	**0.01**
1–3 falls (1 pt)	34 (18.4)	14 (30.2)	20 (14.5)	
4 or more (2 pt)	32 (16.9)	12 (25.6)	20 (14.5)	

**Fig. 1:**
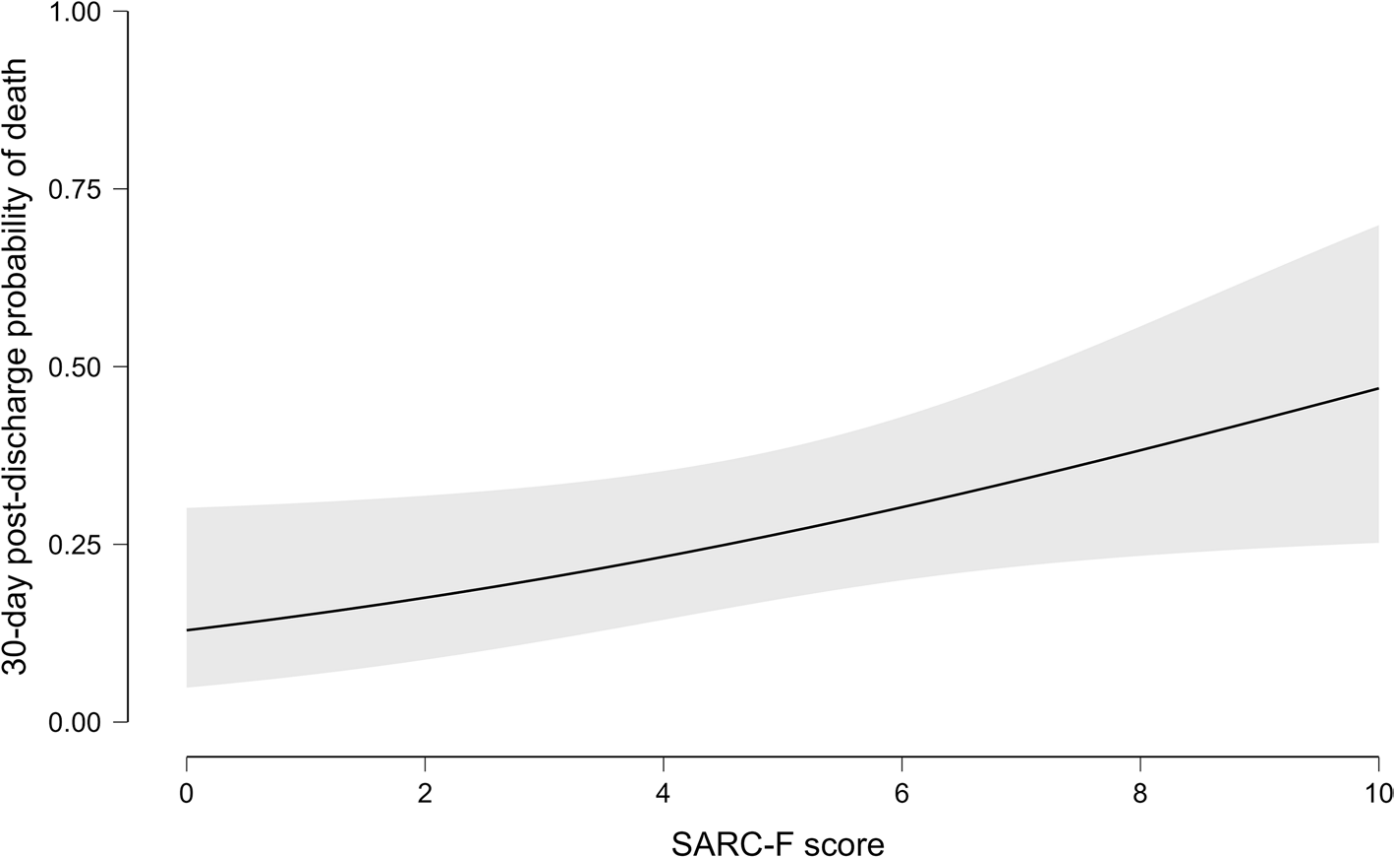
Association between SARC-F and predicted 30-day post-discharge mortality. Multivariable analysis, inferential plot

Using a univariable logistic regression model, SARC-F and SBP were found to be the only statistically significant variables in terms of 30-day post-discharge mortality (see Supplemental Table 7). By multivariable logistic analysis, SARC-F resulted independently associated with mortality after adjustment for age, sex, number of comorbidities, NT-pro-BNP, and SBP at discharge [adjusted OR: 1.13 (CI95% 1.03–1.33), *p* = 0.03], (Fig. 1 and Supplementary Table 2).

**Fig. 2:**
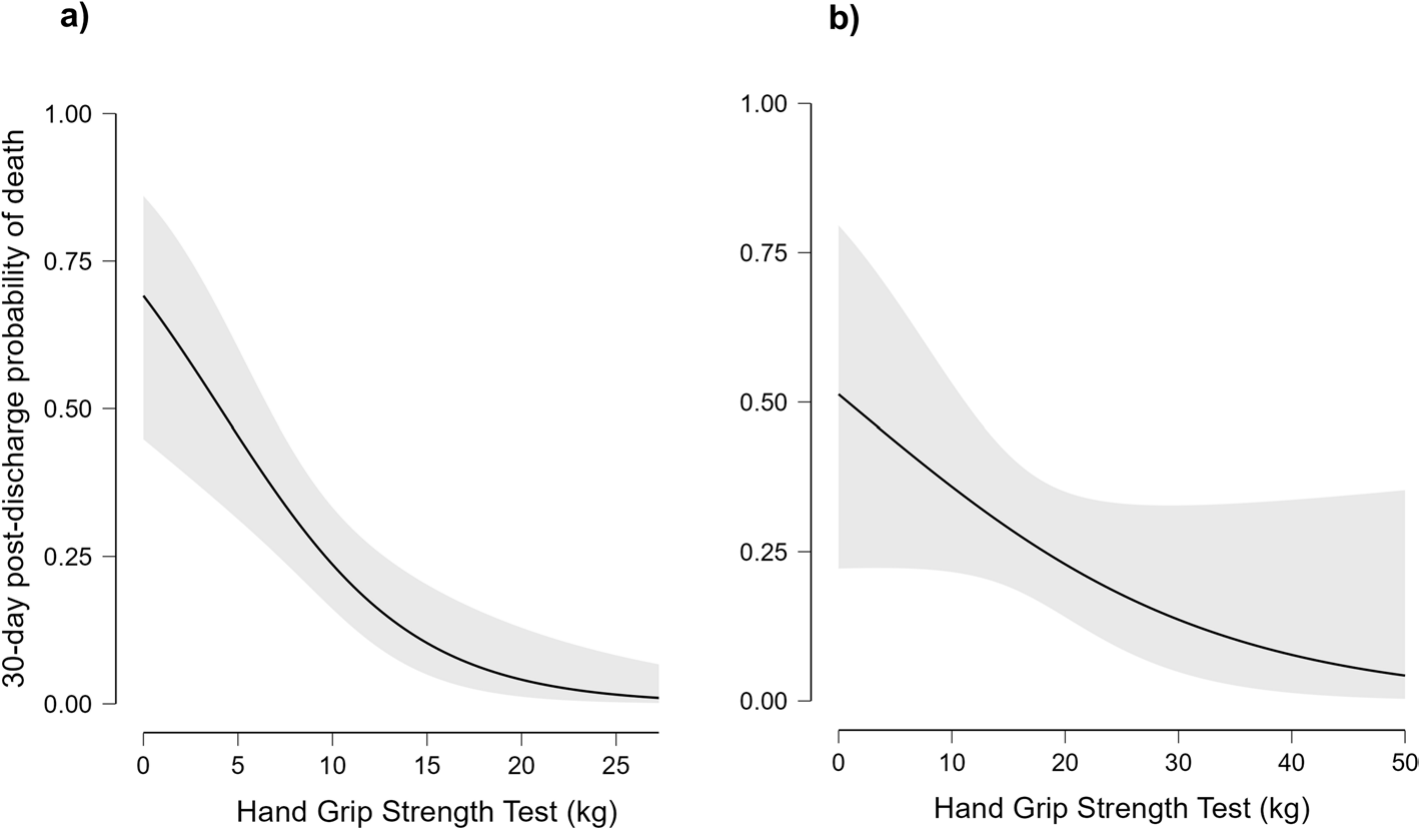
Association between Hand grip strength test and predicted 30-day post-discharge mortality in female (**a**) and male (**b**) patients

Furthermore, HGS score was independently associated with mortality after extensive adjustment for potential confounders [(adjusted standardized *β* = – 0.73 ± 0.03, *p* = 0.008), Fig. 2]. As a secondary analysis, we performed multivariate logistic regression with the SARC-F sub-items (Table 4). The inability to rise from a chair was independently associated with mortality [adjusted OR: 3.26 (CI 95% 1.27–8.34), *p* = 0.014] as well as the number of falls in the last year. More in-depth, both Score 1 (i.e., a number of falls between one and three in the last year) and Score 2 (greater than or equal to four falls) results were independently associated with 30-days mortality [adjusted OR: 3.47 (CI95% 1.28–8.70), *p* = 0.008; adjusted OR: 3.30 (CI 95% 1.28–8.49), *p* = 0.01; respectively (See Supplementary Table 3 and 4]. Finally, by Spearman’s rank correlation, we found a negative correlation between SARC-F values and HGS (Rho = -0.43, *p* < 0.001, Supplementary Fig. 2).

**Table 4:** Relationship between SARC-F and its components with 30-day post-discharge mortality. Multivariable logistic regression

	Odds ratio	95% Confidence interval	*P* value
SARC-F Unadjusted (U)	1.18	1.05–1.34	0.005
SARC-F Adjusted (A)	1.13	1.03–1.33	0.03
Absence of inability on rising from a chair	–	–	–
Inability on rising from a chair (U)	3.34	1.30–8.43	0.009
Inability on rising from a chair (A)	3.26	1.27–8.34	0.014
No falls ref	–	–	–
1–3 falls in the last year (U)	3.25	1.38–7.63	0.007
1–3 falls in the last year (A)	3.47	1.28–8.70	0.008
Greater than or equal to four falls (U)	2.90	1.19–7.68	0.019
Greater than or equal to four falls (A)	3.30	1.29–8.49	0.01

Adjusted model: Age + sex + Systemic Arterial Pressure and NT-pro-BNP at discharge

*Ref* reference

## Discussion

In our prospective, single-center study, we demonstrated the utility of the SARC-F questionnaire and handgrip strength test in predicting 30-day post-discharge mortality among patients aged 75 years and older hospitalized with acutely decompensated HF. For every 1-unit increase in the SARC-F questionnaire, the odds of 30-day mortality rose independently by 18%, a relationship robust to multiple adjustments for confounding variables. This research represents a pioneering attempt to validate a brief sarcopenia risk screening and dynapenia evaluation as a prognostic indicator in the very old patient admitted for acute decompensated heart failure.

In the present study, more than 66% of older patients with acutely decompensated HF were found to be at high risk for sarcopenia, in agreement with a previous study by Zhao et al. [21]. As expected, patients with SARC-F ≥ 4 were older and frailer, showing an 18% higher prevalence of chronic HF as compared to those with SARC-F < 4. This result is consistent with a recent meta-analysis highlighting how patients with heart failure are typically prone to experience sarcopenia [32]. As a fact, sarcopenia and chronic HF seem to strictly have linked one another. In patients with HF, elevated oxidative processes, higher apoptotic activity and the decreased synthesis of striated muscle growth factors contribute to muscle wasting by catabolic shift in muscle homeostasis [6, 33]. Significantly, patients with a SARC-F score of ≥ 4 exhibited heightened pulmonary congestion and higher levels of NT-pro-BNP indicating severe cardiac failure, regardless of hypoalbuminemia status upon hospital admission, in agreement with a recent study[34]. Notably, within the SARC-F sub-items, experiencing one or more falls in the preceding year and complete inability to rise from a chair emerged as robust independent predictors of 30-day mortality, reaffirming the adverse prognostic implications of accidental falls and physical frailty in the oldest-old patient demographic.[35, 36].

Based on our data analysis, we found a direct correlation between lower HGS and increased mortality rates, highlighting HGS as an independent predictor of short-term adverse outcomes in patients with HF, consistent with findings from recent studies [14, 37]. This finding is significant, as it might be reasonable to assume that during the acute phase of hospitalization for HF, HGS may not necessarily reflect muscle weakness. In fact, nearly 80% of patients demonstrated handgrip strength (HGS) values below the normal range, underscoring that the acute phase of illness during hospitalization may not be optimal for defining dynapenia. However, these data suggest that incorporating the HGS test during hospitalization and assessing the SARC-F questionnaire could have notable implications in clinical practice. Not only can it aid in evaluating sarcopenia risk, but it can also contribute to the management and risk stratification of older patients recently discharged for acute HF. International consensus guidelines have recommended identifying frailty during routine clinical encounters by the General Practitioner (GP) or through broader population screening to improve the planning and delivery of services for older adults. However, several obstacles hinder these recommendations, including the need for additional clinical resources and the inaccuracy of simple screening tools. Electronic health record (EHR) systems, such as the electronic Frailty Index [38] (eFI) in the UK and the Primary Care Frailty Index (PC-FI) [39] in Italy, have proven useful in predicting adverse outcomes using routinely collected EHR data. Incorporating the Hand Grip Strength test and assessing its changes after hospital stays could further enhance patient prognosis monitoring in the primary care setting and stratify patients who are most at risk of adverse events following hospital discharge.

Noteworthy, Konishi et al. observed in their multicentre prospective cohort study, that in older patients with heart failure, sarcopenia and low hand grip values significantly affect mortality equally in HFpEF and HFrEF [18]. However, bioelectrical impedance analysis (BIA) or dual-energy X-ray absorptiometry (DEXA) may not always be economically feasible for developing countries or secondary care hospitals. In contrast, the SARC-F questionnaire and handgrip strength test are simple, rapid, and cost-effective tools. Supporting this hypothesis, we demonstrated a statistically significant negative correlation between handgrip strength and SARC-F scores; in other words, higher SARC-F scores are associated with lower handgrip strength. Therefore, beyond the recommended cutoff of 4, the SARC-F may be used as a proxy for dynapenia in settings where more comprehensive assessments are not feasible.

Moreover, our study underscores the potential significance of preventive interventions for patients with acute HF who exhibit an inability to rise from a chair and/or have a history of falls. These individuals may derive substantial benefits from early implementation of functional rehabilitation programs, nutritional reassessment, and evaluation in specialized heart failure outpatient clinics. Significantly, our findings underscore the significance of sarcopenia screening, as identified through these interventions, highlighting its potential reversibility. The Nutritional Intervention Program in Malnourished Patients Admitted for Heart Failure (PICNIC) has demonstrated that personalized nutritional interventions during and after hospitalization for acute HF may confer prognostic benefits [40]. Moreover, a multimodal, multidisciplinary assessment, based on physical activity and dietary recommendation has been proven to be effective in reducing poor outcomes in vulnerable older people [41], with women experiencing greater benefits than men [42, 43].

However, a number of limitations need to be acknowledged. Our study's findings are influenced by the specific patient demographics, healthcare practices, and follow-up protocols of our region. In other settings, these factors might differ significantly, affecting the applicability of our results. For instance, some institutions might have more intensive or prolonged follow-up strategies compared to our center, which could result in different outcomes. The variability in structured follow-up programs across different centers is an important consideration. Some healthcare facilities may offer comprehensive and continuous follow-up, while others may have more limited resources, potentially impacting patient outcomes post-discharge. These differences underscore the need for caution when generalizing our findings to broader settings. To address these limitations, we propose that our findings could be implemented in a variety of healthcare settings with appropriate customization. Institutions could integrate SARC-F and HGS measurements into their routine discharge planning and follow-up programs to identify patients at risk of sarcopenia/dynapenia and associated adverse outcomes. However, it is crucial to tailor these implementations to align with local healthcare practices, available resources, and patient populations to ensure their effectiveness and relevance.

Moreover, the current study has only examined patients at risk for sarcopenia while a definitive sarcopenia diagnosis was not ascertained due to the lack of a validated diagnostic muscle mass assessment; however, muscle mass, strength, and function have been recognized to be strongly influenced by demographic and anthropometric features and standard, uniformed threshold values have not been established [6]. Additionally, the current research was not specifically designed to evaluate sarcopenic patients but to test the prognostic usefulness of a brief sarcopenia and dynapenia assessment.

In conclusion, in the present study increasing SARC-F total score, inability to rise from a chair, history of falls and low HGS score were independently associated with higher short-term mortality risk, thus defining older patients with acutely decompensated HF at high risk of poor outcome following hospitalization.

## Supplementary Information

Below is the link to the electronic supplementary material.

Supplementary file1 (DOCX 3324 KB)

## Data Availability

The datasets used during the current study are available from the corresponding author on reasonable request.

## References

[1] McDonagh TA, Metra M, Adamo M, Gardner RS, Baumbach A, Böhm M et al (2021) 2021 ESC guidelines for the diagnosis and treatment of acute and chronic heart failure: developed by the Task Force for the diagnosis and treatment of acute and chronic heart failure of the European Society of Cardiology (ESC) With the special contributio. Eur Heart J 42(36):3599–3726 34447992 10.1093/eurheartj/ehab368

[2] Braunwald E (2015) The war against heart failure: the Lancet lecture. Lancet 385(9970):812–824 25467564 10.1016/S0140-6736(14)61889-4

[3] Lloyd-Jones D, Adams RJ, Brown TM, Carnethon M, Dai S, De Simone G et al (2010) Heart disease and stroke statistics–2010 update: a report from the American Heart Association. Circulation 121(7):e46-215 20019324 10.1161/CIRCULATIONAHA.109.192667

[4] McDonagh TA, Metra M, Adamo M, Gardner RS, Baumbach A, Böhm M et al (2021) 2021 ESC guidelines for the diagnosis and treatment of acute and chronic heart failure. Eur Heart J 42(36):3599–3726 34447992 10.1093/eurheartj/ehab368

[5] Zuo X, Li X, Tang K, Zhao R, Wu M, Wang Y et al (2023) Sarcopenia and cardiovascular diseases: a systematic review and meta-analysis. J Cachex Sarcopenia Muscle 14(3):1183–1198. 10.1002/jcsm.13221 10.1002/jcsm.13221 PMC10235887 37002802

[6] Lena A, Anker MS, Springer J (2020) Muscle wasting and sarcopenia in heart failure-the current state of science. Int J Mol Sci 21(18):6549 32911600 10.3390/ijms21186549 PMC7555939

[7] Curcio F, Testa G, Liguori I, Papillo M, Flocco V, Panicara V et al (2020) Sarcopenia and heart failure. Nutrients 12(1):211 31947528 10.3390/nu12010211 PMC7019352

[8] Cruz-Jentoft AJ, Bahat G, Bauer J, Boirie Y, Bruyère O, Cederholm T et al (2019) Sarcopenia: revised European consensus on definition and diagnosis. Age Ageing 48(1):16–31 30312372 10.1093/ageing/afy169 PMC6322506

[9] Springer J, Springer JI, Anker SD (2017) Muscle wasting and sarcopenia in heart failure and beyond: update 2017. ESC Heart Fail 4(4):492–498 29154428 10.1002/ehf2.12237 PMC5695190

[10] Ponikowski P, Voors AA, Anker SD, Bueno H, Cleland JGF, Coats AJS et al (2016) 2016 ESC guidelines for the diagnosis and treatment of acute and chronic heart failure: the Task Force for the diagnosis and treatment of acute and chronic heart failure of the European Society of Cardiology (ESC)—developed with the special contribution. Eur J Heart Fail 18(8):891–975 27207191 10.1002/ejhf.592

[11] Loncar G, Springer J, Anker M, Doehner W, Lainscak M (2016) Cardiac cachexia: hic et nunc. J Cachex Sarcopenia Muscle 7(3):246–260 10.1002/jcsm.12118 PMC4929818 27386168

[12] Chen R, Xu J, Wang Y, Jiang B, Xu X, Lan Y et al (2023) Prevalence of sarcopenia and its association with clinical outcomes in heart failure: an updated meta-analysis and systematic review. Clin Cardiol 46(3):260–268 36644878 10.1002/clc.23970 PMC10018088

[13] Matsue Y, Kamiya K, Saito H, Saito K, Ogasahara Y, Maekawa E et al (2020) Prevalence and prognostic impact of the coexistence of multiple frailty domains in elderly patients with heart failure: the FRAGILE-HF cohort study. Eur J Heart Fail 22(11):2112–2119 32500539 10.1002/ejhf.1926

[14] Prokopidis K, Triantafyllidis KK, Kechagias KS, Mitropoulos A, Sankaranarayanan R, Isanejad M (2023) Are sarcopenia and its individual components linked to all-cause mortality in heart failure? A systematic review and meta-analysis. Clin Res Cardiol. 10.1007/s00392-023-02360-8 38085294 10.1007/s00392-023-02360-8 PMC12058882

[15] Honda S, Uemura Y, Shibata R, Sekino T, Takemoto K, Ishikawa S et al (2022) Clinical implications of severe sarcopenia in Japanese patients with acute heart failure. Geriatr Gerontol Int 22(6):477–482 35460315 10.1111/ggi.14389

[16] Xu J, Reijnierse EM, Pacifico J, Wan CS, Maier AB (2021) Sarcopenia is associated with 3-month and 1-year mortality in geriatric rehabilitation inpatients: RESORT. Age Ageing 50(6):2147–2156 34260683 10.1093/ageing/afab134 PMC8581377

[17] Maeda D, Matsue Y, Kagiyama N, Jujo K, Saito K, Kamiya K et al (2022) Sex differences in the prevalence and prognostic impact of physical frailty and sarcopenia among older patients with heart failure. Nutr Metab Cardiovasc Dis 32(2):365–372 34893406 10.1016/j.numecd.2021.10.012

[18] Konishi M, Kagiyama N, Kamiya K, Saito H, Saito K, Ogasahara Y et al (2021) Impact of sarcopenia on prognosis in patients with heart failure with reduced and preserved ejection fraction. Eur J Prev Cardiol 28(9):1022–1029 33624112 10.1093/eurjpc/zwaa117

[19] Guralnik JM, Simonsick EM, Ferrucci L, Glynn RJ, Berkman LF, Blazer DG et al (1994) A short physical performance battery assessing lower extremity function: association with self-reported disability and prediction of mortality and nursing home admission. J Gerontol 49(2):M85-94 8126356 10.1093/geronj/49.2.m85

[20] Tanaka S, Kamiya K, Hamazaki N, Matsuzawa R, Nozaki K, Maekawa E et al (2017) Utility of SARC-F for assessing physical function in elderly patients with cardiovascular disease. J Am Med Dir Assoc 18(2):176–181 28043805 10.1016/j.jamda.2016.10.019

[21] Zhao W, Lu M, Wang X, Guo Y (2021) The role of sarcopenia questionnaires in hospitalized patients with chronic heart failure. Aging Clin Exp Res 33(2):339–344 32346826 10.1007/s40520-020-01561-9 PMC7914185

[22] Nagre A (2019) Focus-assessed transthoracic echocardiography: implications in perioperative and intensive care. Ann Cardiac Anaesth 22(3):302–308 10.4103/aca.ACA_88_18 PMC6639886 31274494

[23] Lindner M, Thomas R, Claggett B, Lewis EF, Groarke J, Merz AA et al (2020) Quantification of pleural effusions on thoracic ultrasound in acute heart failure. Eur Heart J Acute Cardiovasc Care 9(5):513–521 31976745 10.1177/2048872620901835 PMC7644143

[24] Stuck AE, Siu AL, Wieland GD, Adams J, Rubenstein LZ (1993) Comprehensive geriatric assessment: a meta-analysis of controlled trials. Lancet 342(8878):1032–1036 8105269 10.1016/0140-6736(93)92884-v

[25] Pfeiffer E (1975) A short portable mental status questionnaire for the assessment of organic brain deficit in elderly patients. J Am Geriatr Soc 23(10):433–441 1159263 10.1111/j.1532-5415.1975.tb00927.x

[26] Katz S, Ford A, Moskowitz R, Jackson B, Jaffe M (1963) Studies of illness in the aged: the index of ADL: a standardized measure of biological and psychosocial function. JAMA 185:914–919 14044222 10.1001/jama.1963.03060120024016

[27] Lawton MP, Brody EM (1969) Assessment of older people: self-maintaining and instrumental activities of daily living. Gerontologist 9(3):179–186 5349366

[28] Theou O, Pérez-Zepeda MU, van der Valk AM, Searle SD, Howlett SE, Rockwood K (2021) A classification tree to assist with routine scoring of the clinical frailty scale. Age Ageing 50(4):1406–1411 33605412 10.1093/ageing/afab006 PMC7929455

[29] Guigoz Y, Lauque S, Vellas BJ (2002) Identifying the elderly at risk for malnutrition: the mini nutritional assessment. Clin Geriatr Med 18(4):737–757 12608501 10.1016/s0749-0690(02)00059-9

[30] Barbosa-Silva TG, Menezes AMB, Bielemann RM, Malmstrom TK, Gonzalez MC (2016) Enhancing SARC-F: improving sarcopenia screening in the clinical practice. J Am Med Dir Assoc 17(12):1136–1141 27650212 10.1016/j.jamda.2016.08.004

[31] Okoye C, Mazzarone T, Niccolai F, Bencivenga L, Pescatore G, Bianco MG et al (2023) Predicting mortality and re-hospitalization for heart failure: a machine-learning and cluster analysis on frailty and comorbidity. Aging Clin Exp Res 35(12):2919 37848804 10.1007/s40520-023-02566-w PMC10721693

[32] Zhang Y, Zhang J, Ni W, Yuan X, Zhang H, Li P et al (2021) Sarcopenia in heart failure: a systematic review and meta-analysis. ESC Heart Fail 8(2):1007–1017 33576177 10.1002/ehf2.13255 PMC8006658

[33] Castiglione V, Gentile F, Vergaro G (2023) Cachexia, sarcopenia and heart failure: a last mile to be walked. Int J Cardiol 388:131131 37364716 10.1016/j.ijcard.2023.131131

[34] Prokopidis K, Morwani-Mangnani J, McDowell G, Lip GYH, Venturelli M, Sankaranarayanan R et al (2024) Sarcopenia is linked to higher levels of B-type natriuretic peptide and its N-terminal fragment in heart failure: a systematic review and meta-analysis. Eur Geriatr Med 15(4):893–901 38457043 10.1007/s41999-024-00950-x PMC11377361

[35] Hartholt KA, van Beeck EF, van der Cammen TJM (2018) Mortality from falls in Dutch adults 80 years and older, 2000–2016. JAMA 319(13):1380–1382 29614170 10.1001/jama.2018.1444 PMC5933376

[36] Florence CS, Bergen G, Atherly A, Burns E, Stevens J, Drake C (2018) Medical costs of fatal and nonfatal falls in older adults. J Am Geriatr Soc 66(4):693–698 29512120 10.1111/jgs.15304 PMC6089380

[37] Charkiewicz M, Wojszel ZB, Kasiukiewicz A, Magnuszewski L, Wojszel A (2023) Association of chronic heart failure with frailty, malnutrition, and sarcopenia parameters in older patients-a cross-sectional study in a geriatric ward. J Clin Med. 12(6):2305 36983305 10.3390/jcm12062305 PMC10052656

[38] Clegg A, Bates C, Young J, Ryan R, Nichols L, Ann Teale E et al (2016) Development and validation of an electronic frailty index using routine primary care electronic health record data. Age Ageing 45(3):353–360 26944937 10.1093/ageing/afw039 PMC4846793

[39] Vetrano DL, Zucchelli A, Onder G, Fratiglioni L, Calderón-Larrañaga A, Marengoni A et al (2023) Frailty detection among primary care older patients through the Primary Care Frailty Index (PC-FI). Sci Rep 13(1):3543 36864098 10.1038/s41598-023-30350-3 PMC9981758

[40] Toth MJ, Gottlieb SS, Goran MI, Fisher ML, Poehlman ET (1997) Daily energy expenditure in free-living heart failure patients. Am J Physiol-Endocrinol Metabol 272(3):E469–E475 10.1152/ajpendo.1997.272.3.E469 9124554

[41] Bernabei R, Landi F, Calvani R, Cesari M, Del Signore S, Anker SD et al (2022) Multicomponent intervention to prevent mobility disability in frail older adults: randomised controlled trial (SPRINTT project). BMJ 377:e068788 35545258 10.1136/bmj-2021-068788 PMC9092831

[42] Zhang Y, Zou L, Chen ST, Bae JH, Kim DY, Liu X et al (2021) Effects and moderators of exercise on sarcopenic components in sarcopenic elderly: a systematic review and meta-analysis. Front Med 8:649748 10.3389/fmed.2021.649748 PMC8169963 34095166

[43] Chen N, He X, Feng Y, Ainsworth BE, Liu Y (2021) Effects of resistance training in healthy older people with sarcopenia: a systematic review and meta-analysis of randomized controlled trials. Eur Rev Aging Phys Act 18(1):23 34763651 10.1186/s11556-021-00277-7 PMC8588688

