# Supplementary material for: Usefulness of the SARC-F questionnaire and the measurement of the hand grip strength in predicting short-term mortality in older patients hospitalized for acute heart failure
Source: Eur Geriatr Med. 2024 Sep 27;15(6):1839–47. doi: 10.1007/s41999-024-01054-2 (PMC11631818; doi:10.1007/s41999-024-01054-2)
Supplement: Supplementary file 1 — Supplementary file1 (DOCX 3324 KB) [file 41999_2024_1054_MOESM1_ESM.docx]

**Supplementary Figure 1: Study Flowchart**

**
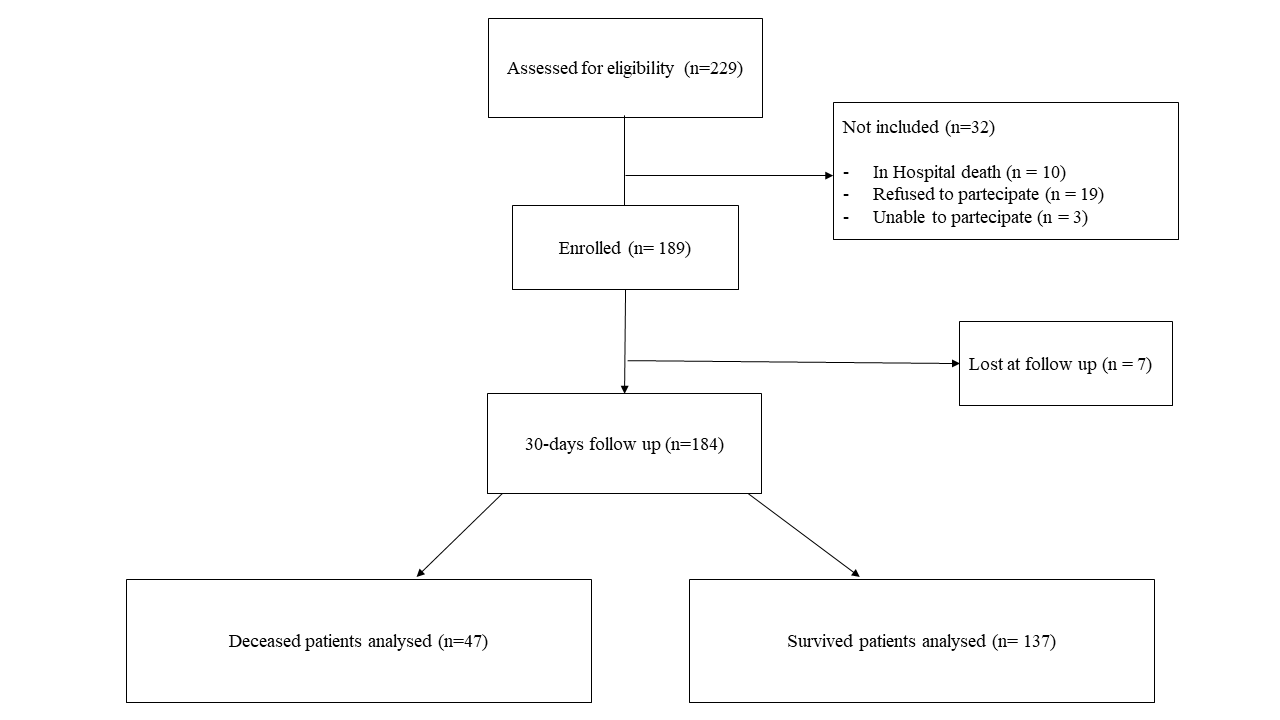
**

**Supplementary Figure 2: Correlation plot between Hand Grip Test and SARC-F values.**


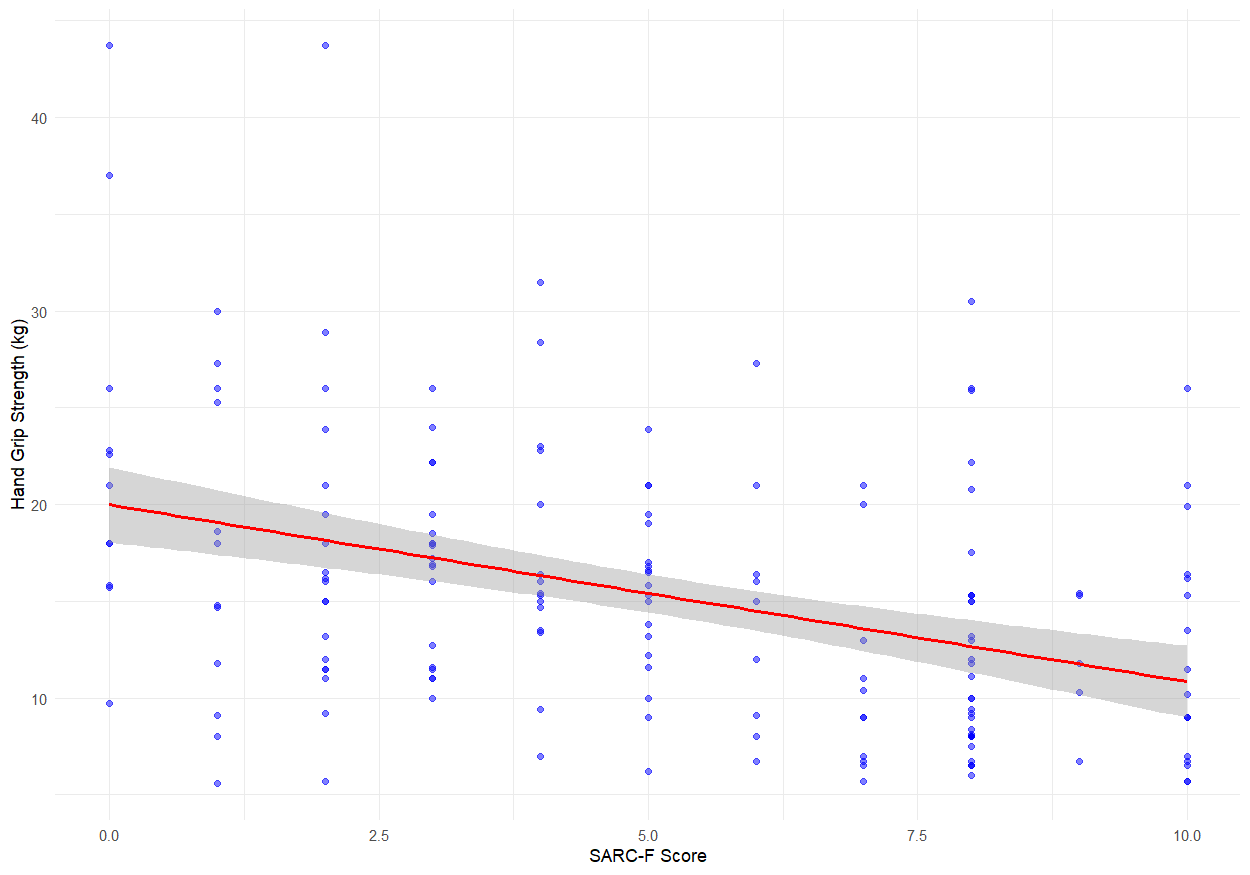


**Supplementary Table 1: Characteristics of study population in dead and alive patients (1)**

|  | All patients  N=184 | Dead  N=47 | Alive  N=137 | P-value |
| --- | --- | --- | --- | --- |
| Comorbidities | | | | |
| COPD (%) | 47 (25.9) | 12 (26.1) | 35 (25.9) | 0.98 |
| Hypertension (%) | 120 (65.5) | 25 (54) | 95 (69) | 0.06 |
| Chronic Heart Failure (%) | 116 (63) | 34 (74) | 82 (60) | 0.08 |
| Diabetes (%) | 52 (28.8) | 12 (26.1) | 40 (29.8) | 0.63 |
| Stroke (%) | 32 (17.5) | 12 (26.1) | 20 (14.5) | 0.17 |
| Chronic renal failure (%) | 60 (32.8) | 20 (43.5) | 40 (29) | 0.07 |
| Ischemic heart disease (%) | 65 (35.6) | 19 (41.3) | 46 (33.6) | 0.35 |
| Atrial fibrillation (%) | 116 (63.3) | 32 (67.4) | 84 (61.8) | 0.50 |
| Cancer (%) | 39 (21.5) | 11 (23.9) | 28 (20.6) | 0.64 |
| Lung ultrasound and FOCUS measurements | | | | |
| B-lines number median (IQR) | 12 (12) | 12 (11.5) | 12 (13) | 0.77 |
| PEFS cumulative median (IQR) | 2 (5) | 4 (4.5) | 2 (5) | 0.13 |
| IVC max mean, mm (SD) | 17.3 (5.6) | 17.9 (6) | 17.1 (5.5) | 0.49 |
| IVC min mean, mm (SD) | 10.6 (6.9) | 11.6 (7.7) | 10.3 (6.7) | 0.35 |
| LVEF < 40% (%) | 58 (31.7) | 15 (31.4) | 43 (31.8) | 0.97 |
| LVEF %, median | 52(10) | 53(17.5) | 52(15.5) | 0.68 |
| Mitral valve failure  Mild  Moderate  Severe | 89 (48.3)  40 (27.2)  39 (26.3)  10 (6.7) | 7 (19.4)  16 (44.4)  3 (8.3) | 33 (29.4)  23 (20.5)  7 (6.2) | <0.001 |
| Mitral valve stenosis  Mild  Moderate | 6 (3.2)  4 (2.7)  2 (1.3) | 0(0.0)  0(0.0) | 4 (3.5)  2 (1.7) | 0.3 |
| Aortic valve failure  Mild  Moderate | 45(24.4)  36 (24.3)  9 (6.0) | 13 (36.1)  2 (5.5) | 23 (20.5)  7 (6.2) | <0.001 |
| Aortic valve stenosis  Mild  Moderate  Severe | 21(11.4)  13 (8.7)  3 (2.0)  5 (3.3) | 5 (13.8)  0 (0.0)  2 (5.5) | 8 (6.5)  3 (2.4)  3 (2.4) | 0.06 |
| Tricuspidalic valve failure  Mild  Moderate  Severe | 61(33.1)  39 (26.3)  15(10.1)  7 (4.7) | 11 (30.5)  6 (16.7)  1 (2.7) | 28 (25.0)  9 (8.0)  6 (5.3) | 0.01 |

**Supplementary Table 2: Characteristics of study population in dead and alive patients (2)**

|  | All patients  N=184 | Dead  N=47 | Alive  N=137 | P-value |
| --- | --- | --- | --- | --- |
| Blood exams |  |  |  |  |
| Mean Creatinine (SD) | 1.39 (0.71) | 1.49(0.84) | 1.36(0.65) | 0.395 |
| Albumin mean, g/dl (SD) | 3.03 (0.53) | 2.9 (0.55) | 3.1 (0.51) | 0.19 |
| Mean Hemoglobin g/L (SD) | 10.7(1.8) | 10.4(1.9) | 10.8(1.7) | 0.318 |
| P/F admission mean (SD) | 311.3 (93.7) | 308.3 (100.5) | 312.3 (91.6) | 0.80 |
| Medications | | | | |
| Number of medications | 8(3) | 8(3) | 9(3) | 0.60 |
| Beta-blockers at discharge (%) | 151 (82.2) | 40 (84.2) | 111 (81.3) | 0.78 |
| ACE-I at discharge (%) | 71 (38.7) | 23 (47.3) | 48 (34.8) | 0.35 |
| ARB at discharge (%) | 18 (9.6) | 5 (10.5) | 13 (9.3) | 0.88 |
| MRA at discharge (%) | 32 (17.7) | 4 (10.5) | 28 (21) | 0.32 |
| Furosemide at discharge (%) | 151 (82.2) | 42 (89.4) | 109 (79) | 0.32 |
| Digoxin at discharge (%) | 15 (8) | 0 (0) | 15 (11.6) | 0.12 |
| SGLT2-i at discharge (%) | 9 (4.8) | 0 (0) | 9 (6.9) | 0.23 |
| Statins (%) | 106 | 26(57%) | 80(58%) | 0.78 |
| Outcomes | | | | |
| Upgrade diuretic therapy (%) | 75 (41) | 15 (32.5) | 60 (43.8) | 0.20 |
| Pulmonary edema (%) | 26 (14.1) | 7 (15) | 19 (13.8) | 0.85 |
| Adverse events (%) | 53 (28.9) | 16 (35) | 37 (27.1) | 0.34 |
| Post-discharge events |  |  |  |  |
| 30 days readmission (%) | 25 (13.9) | 5 (11.4) | 20 (14.8) | 0.62 |

Mann-Whitney test, Pearson’s Chi-squared test or Fisher Test as appropriate. P-values <0.05 are in bold.

*Abbreviations:* ACE-i, Angiotensin-Converting Enzyme inhibitors; ARB, Angiotensin Receptor Blocker; COPD, Chronic Obstructive Pulmonary Disease; HR, Heart Rate; IVC, Inferior Vena Cava; LVEF, Left Ventricular Ejection Fraction; MRA, Mineralocorticoid Receptor Antagonist; NT-proBNP, N-Terminal pro-B-type Natriuretic Peptide; P/F, PaO2/FiO2 ratio; PEFS, Pleural EFfusion Score; SARC-F, Strength- Assistance with walking- Rising from a chair- Climbing stairs and Falls questionnaire; SBP, Systolic Blood Pressure; SGLT2-i, Sodium-GLucose coTransporter-2 inhibitors

**Supplementary Table 3: Characteristics of study population according to Hand grip strength test values (HSG) among male individuals**

| **Variable** | HSG < 27, N = 63*^1^* | HSG ≥ 27, N = 10*^1^* | **p-value***^2^* |
| --- | --- | --- | --- |
| **Age mean, years (SD)** | 87.0 (83.0, 90.0) | 84.0 (83.3, 85.0) | 0.020 |
| **BMI mean, kg/m^2^ (SD)** | 24.0 (21.9, 27.0) | 26.4 (24.6, 30.2) | 0.071 |
| **SARC-F median (IQR)** | 5.0 (2.0, 8.0) | 2.0 (1.0, 3.5) | 0.027 |
| **Number of comorbidities , median (IQR)** | 4.00 (3.00, 6.00) | 4.00 (4.00, 4.75) | 0.83 |
| **LVEF < 40% (%)** | 29 (59%) | 2 (40%) | 0.65 |
| **Mean SBP (SD)** | 118 (105, 136) | 127 (119, 136) | 0.62 |
| **NT-proBNP discharge median, pg/ml (IQR)** | 7,704 (4,661, 15,319) | 2,664 (954, 6,566) | 0.009 |
| **Median Creatinine (IQR)** | 1.28 (0.92, 1.76) | 1.63 (1.33, 1.93) | 0.76 |
| **Mean Hemoglobin g/L (SD)** | 10.80 (9.40, 11.70) | 12.90 (12.40, 13.40) | 0.10 |
| **30-day mortality (%)** | 18 (29%) | 1 (10%) | 0.45 |
| Median (IQR); n (%)*^2^* Wilcoxon rank sum test; Pearson’s Chi-squared test | | | |

*:*

*Abbreviations***:** BMI indicates Body Mass Index; LVEF, Left Ventricular Ejection Fraction; NT-proBNP, N-Terminal pro-B-type Natriuretic Peptide; SBP, Systolic Blood Pressure

**Supplementary Table 4: Characteristics of study population according to Hand grip strength test values (HSG) among female individuals**

| **Variable** | HSG < 27, N = 92*^1^* | HSG ≥ 27, N = 27*^1^* | **p-value***^2^* |
| --- | --- | --- | --- |
| **Age mean, years (SD)** | 88.5 (84.8, 92.0) | 84.0 (80.5, 91.0) | 0.045 |
| **BMI mean, kg/m^2^ (SD)** | 24.0 (20.3, 27.0) | 23.0 (21.9, 28.0) | 0.78 |
| **SARC-F median (IQR)** | 7.00 (4.00, 8.00) | 3.00 (3.00, 6.00) | 0.010 |
| **# comorbidities , median (IQR)** | 4.00 (3.00, 5.00) | 2.00 (2.00, 5.00) | 0.030 |
| **LVEF < 40% (%)** | 56 (79%) | 13 (54%) | 0.019 |
| **Mean SBP (SD)** | 134 (110, 150) | 130 (105, 130) | 0.15 |
| **NT-proBNP discharge median, pg/ml (IQR)** | 10,230 (4,965, 25,789) | 7,547 (4,032, 11,145) | 0.076 |
| **Median Creatinine (IQR)** | 1.40 (0.93, 1.71) | 1.17 (0.97, 1.58) | 0.77 |
| **Mean Hemoglobin g/L (SD)** | 10.00 (9.18, 11.43) | 11.50 (10.28, 12.40) | 0.14 |
| **30-day mortality (%)** | 25 (27%) | 3 (11%) | 0.079 |
| Median (IQR); n (%)*^2^* Wilcoxon rank sum test; Pearson’s Chi-squared test | | | |

*Abbreviations***:** BMI indicates Body Mass Index; LVEF, Left Ventricular Ejection Fraction; NT-proBNP, N-Terminal pro-B-type Natriuretic Peptide; SBP, Systolic Blood Pressure

**Supplementary Table 5: Characteristics of study population according to SARC-F values (1)**

|  | All patients  N=184 | SARC-F ≥4  N=122 | SARC-F < 4  N=62 | P-value |
| --- | --- | --- | --- | --- |
| Comorbidities |  |  |  |  |
| COPD (%) | 47 (25.9) | 30 (25.4) | 17 (28.8) | 0.63*^2^* |
| Hypertension (%) | 120 (65.5) | 82 (67.5) | 38 (62.7) | 0.52*^2^* |
| Chronic Heart Failure (%) | 116 (63) | 84 (68.4) | 32 (50.8) | **0.02***^2^* |
| Diabetes (%) | 52 (28.8) | 34 (27.2) | 18 (28.9) | 0.82*^2^* |
| Stroke (%) | 32 (17.5) | 25 (21) | 7 (11.9) | 0.13*^2^* |
| Chronic renal failure (%) | 60 (32.8) | 43 (35) | 17 (27.1) | 0.29*^2^* |
| Ischemic heart disease (%) | 65 (35.6) | 44 (35.9) | 21 (32.2) | 0.62*^2^* |
| Atrial fibrillation (%) | 116 (63.3) | 80 (65.8) | 36 (57.7) | 0.30*^2^* |
| Cancer (%) | 39 (21.5) | 24 (20.2) | 15 (25.4) | 0.42*^2^* |
| Lung ultrasound and FOCUS measurements | | | | |
| B-lines number median (IQR) | 12 (12) | 11.5 (12) | 9.5 (13.75) | 0.75*^2^* |
| PEFS cumulative median (IQR) | 2 (5) | 3 (5) | 1 (4) | **0.009***^1^* |
| IVC max mean, mm (SD) | 17.3 (5.6) | 16.9 (6) | 18.2 (5) | 0.17*^1^* |
| IVC min mean, mm (SD) | 10.6 (6.9) | 10.6 (7.1) | 10.7 (6.9) | 0.95*^1^* |
| LVEF < 40% (%) | 58 (31.7) | 25 (28) | 15 (35) | 0.36*^2^* |
| LVEF %, median | 52(10) | 53(18) | 50(15) | 0.36 |
| Mitral insufficiency  Mild  Moderate  Severe | 89 (48.3)  41 (28.0)  31 (26.0)  10 (6.7) | 32 (31.6)  23 (23.4)  5 (5.1) | 9 (18.0)  15 (30.0)  5 (10.0) | 0.24 |
| Mitral stenosis  Mild  Moderate | 6 (3.2)  4 (2.7)  2 (0.6) | 3 (3.1)  1 (1.0) | 1 (2.0)  1 (2.0) | 0.41 |
| Aortic valve failure  Mild  Moderate | 45(24.4)  36 (30.4)  5 (6.7) | 32 (35.7)  3 (2.0) | 3 (20.0)  2 (4.0) | 0.28 |
| Aortic valve stenosis  Mild  Moderate  Severe | 21(11.4)  13 (12.1)  3 (2.7)  5 (4.7) | 10 (10.2)  2 (1.1)  3 (3.0) | 3 (6.0)  1 (6.0)  2 (4.0) | 0.20 |
| Tricuspidal valve failure  Mild  Moderate  Severe | 61(33.1)  39 (26.3)  15 (10.1)  7 (4.7) | 12(12.2)  4 (4.1)  2 (2.0) | 27 (54.0)  11 (22.0)  5 (10.0) | 0.10 |

**Supplementary Table 6: Characteristics of study population according to SARC-F values (2)**

|  | All patients  N=184 | SARC-F ≥4  N=122 | SARC-F < 4  N=62 | P-value |
| --- | --- | --- | --- | --- |
| Blood exams |  |  |  |  |
| Mean Creatinine (SD) | 1.39 (0.71) | 1.45 (0.75) | 1,26 (0.59) | 0.21*^1^* |
| Mean Hemoglobin g/L (SD) | 10.7 (1.8) | 10.5 (1.8) | 11.3 (1.7) | 0.05*^1^* |
| P/F admission mean (SD) | 311.3 (93.7) | 316.3 (90.3) | 298.9 (101.4) | 0.26*^1^* |
| HCO3- admission mean, mmol/L (SD) | 25.4 (4.4) | 25.7 (4.7) | 25.1 (3.7) | 0.43*^1^* |
| HCO3- dimission mean, mmol/L (SD) | 29.3 (5.7) | 29.5 (6.2) | 28.8 (4.4) | 0.45*^1^* |
| Medications | | | | |
| Number of medications, median (IQR) | 8(3) | 9(4) | 8(4) | 0.11 |
| Beta-blockers at discharge (%) | 151 (82.2) | 108 (88.3) | 43 (68.4) | 0.06 |
| ACE-i at discharge (%) | 71 (38.7) | 42 (34.8) | 29 (47.3) | 0.35 |
| ARB at discharge (%) | 18 (9.6) | 8 (6.9) | 10 (15.7) | 0.27 |
| MRA at discharge (%) | 32 (17.7) | 26 (21) | 6 (10.5) | 0.32 |
| Furosemide at discharge (%) | 151 (82.2) | 102 (83.7) | 49 (79) | 0.65 |
| Digoxin at discharge (%) | 15 (8) | 8 (7) | 7 (10.5) | 0.63 |
| SGLT2-i at discharge (%) | 9 (4.8) | 3 (2.3) | 6 (10.5) | 0.16 |
| Statins | 106 | 67 (54%) | 39 (63%) | 0.33 |
| In-Hospital events |  |  |  |  |
| Hospital stay median, days (IQR) | 7 (3) | 7 (3) | 7 (3.25) | 0.60*^1^* |
| Upgrade diuretic therapy (%) | 75 (41) | 49 (39.6) | 26 (42.1) | 0.75*^2^* |
| Pulmonary edema (%) | 26 (14.1) | 17 (14.4) | 9 (14) | 0.95*^2^* |
| Adverse events (%) | 53 (28.9) | 38 (31) | 15 (24.6) | 0.40*^2^* |
| Post-discharge events |  |  |  |  |
| 30 days readmission (%) | 25 (13.9) | 16 (13.4) | 9 (15.2) | 0.77*^2^* |
| 30 days mortality (%) | 47 (25.5) | 37 (30) | 10 (16) | **0.04***^2^* |

Mann-Whitney test, Pearson’s Chi-squared test or Fisher Test as appropriate. P-values <0.05 are in bold.

*Abbreviations:* ACE-i, Angiotensin-Converting Enzyme inhibitors; ARB, Angiotensin Receptor Blocker; COPD, Chronic Obstructive Pulmonary Disease; HR, Heart Rate; IVC, Inferior Vena Cava; LVEF, Left Ventricular Ejection Fraction; MRA, Mineralocorticoid Receptor Antagonist; NT-proBNP, N-Terminal pro-B-type Natriuretic Peptide; P/F, PaO2/FiO2 ratio; PEFS, Pleural EFfusion Score; SARC-F, Strength- Assistance with walking- Rising from a chair- Climbing stairs and Falls questionnaire; SBP, Systolic Blood Pressure; SGLT2-i, Sodium-GLucose coTransporter-2 inhibitors

**Supplementary Table 7: Logistic multivariable regression. Univariate model.**

|  | Beta Estimate | Standard Error | P-value |
| --- | --- | --- | --- |
| SBP | -0.022 | 0.011 | **0.042** |
| Creatinine | 0.250 | 0.293 | 0.394 |
| NT-pro-BNP | 0.0004 | 0.0001 | **0.001** |
| Sex (F) | 0.030 | 0.345 | 0.931 |
| Age | 0.031 | 0.029 | 0.283 |
| Hemoglobin | -0.124 | 0.124 | 0.316 |
| HR | 0.024 | 0.017 | 0.148 |
| B-lines number | -0.005 | 0.017 | 0.766 |
| BMI | -0.031 | 0.044 | 0.490 |
| SARC-F | 0.171 | 0.062 | **0.005** |
| P/F admission | -0.000 | 0.002 | 0.804 |
| Number of Comorbidities | 0.088 | 0.083 | 0.287 |
| LVEF | 0.030 | 0.419 | 0.943 |

Mann-Whitney test, Pearson’s Chi-squared test or Fisher Test as appropriate. P-values <0.05 are in bold.

*Abbreviations***:**; SBP, Systolic Blood Pressure COPD, Chronic Obstructive Pulmonary Disease; PEFS, Pleural EFfusion Score; IVC, Inferior Vena Cava; LVEF, Left Ventricular Ejection Fraction; NT-proBNP, N-Terminal pro-B-type Natriuretic Peptide; SARC-F, Strength- Assistance with walking- Rising from a chair- Climbing stairs and Falls questionnaire;; HR, Heart Rate; P/F, PaO2/FiO2 ratio; ACE-i, Angiotensin-Converting Enzyme inhibitors; ARB, Angiotensin Receptor Blocker; MRA, Mineralocorticoid Receptor Antagonist; SGLT2-i, Sodium-GLucose coTransporter-2 inhibitors
